# RoPEUS: A New Robust Algorithm for Static Positioning in Ultrasonic Systems

**DOI:** 10.3390/s90604211

**Published:** 2009-06-03

**Authors:** José Carlos Prieto, Christophe Croux, Antonio Ramón Jiménez

**Affiliations:** 1 LOPSI group, Instituto de Automática Industrial, Consejo Superior de Investigaciones Científicas (CSIC), Ctra. Campo Real Km 0.200, 28500 La Poveda-Arganda del Rey, Madrid, Spain; E-mail: arjimenez@iai.csic.es (A.R.J.); 2 Faculty of Business and Economics, K.U.Leuven, Naamsestraat 69, 3000 Leuven, Belgium; E-mail: christophe.croux@econ.kuleuven.be (C.C.)

**Keywords:** local positioning systems, robust positioning, robust statistics, ubiquitous computing

## Abstract

A well known problem for precise positioning in real environments is the presence of outliers in the measurement sample. Its importance is even bigger in ultrasound based systems since this technology needs a direct line of sight between emitters and receivers. Standard techniques for outlier detection in range based systems do not usually employ robust algorithms, failing when multiple outliers are present. The direct application of standard robust regression algorithms fails in static positioning (where only the current measurement sample is considered) in real ultrasound based systems mainly due to the limited number of measurements and the geometry effects. This paper presents a new robust algorithm, called RoPEUS, based on MM estimation, that follows a typical two-step strategy: 1) a high breakdown point algorithm to obtain a clean sample, and 2) a refinement algorithm to increase the accuracy of the solution. The main modifications proposed to the standard MM robust algorithm are a built in check of partial solutions in the first step (rejecting bad geometries) and the off-line calculation of the scale of the measurements. The algorithm is tested with real samples obtained with the 3D-LOCUS ultrasound localization system in an ideal environment without obstacles. These measurements are corrupted with typical outlying patterns to numerically evaluate the algorithm performance with respect to the standard parity space algorithm. The algorithm proves to be robust under single or multiple outliers, providing similar accuracy figures in all cases.

## 1. Introduction

The general implantation of Global Navigation Satellite Systems (GNSS) has triggered the development of new applications based on the user's position. Their limited use in indoor environments [1] has created the necessity for new positioning systems able to localize in environments where GNSS signals are not available.

Several technologies are employed to design custom positioning systems able to work in indoor or restricted environments, referred to as Local Positioning Systems (LPS) [2, 3]. They are commonly classified by their accuracy, which is directly dependent on the technology: systems based on Received Signal Strength Indicator (RSSI) achieve accuracies of several meters [4, 5]; Ultra-Wide Band (UWB) radio systems, using the measurement of Time of Flight (ToF), achieve errors within tens of centimeters [6, 7]; artificial vision navigation systems have accuracies of several centimeters [8]; and finally, ultrasound location systems are able to offer accuracies below one centimeter [9].

A well known problem for precise positioning in real environments is the presence of outliers in the measurement sample. These outliers are provoked by obstacles present in the localization area generating reflected alias signals (multipath effect), or blocking the direct signal path producing distortion in the received signal or even avoiding its reception.

The literature concerning outlier detection in the Global Positioning System (GPS) is extensive, whereas this is not the case for LPSs, especially those using ultrasound. Most of the GPS articles are devoted to the existence of a single faulty measurement, since it was developed for aerial navigation where the presence of two simultaneous faults is very unlikely. However, the tendency is changing to algorithms that detect multiple failures, that are more likely to occur when using GPS in urban areas or, in the close future, in combination with others GNSS. Although GPS has several procedures to detect system errors, we will concentrate on those techniques developed to detect faulty measurements in the receiver (Receiver Autonomous Integrity Monitoring - RAIM), since they can be employed on every localization system based on ranges.

Outlier detection methods can be divided into two main categories: 1) backward search, outliers are detected and removed one at a time; 2) robust methodologies that use forward search, look for an initial clean measurement sample without outliers and add good measurements to improve the result, or robust functions, that are indirect procedures to detect outliers downweighting atypical observations.

The Parity Space (PS) [10] is a backward search method, being the most prominent Receiver Autonomous Integrity Monitoring (RAIM) algorithm. In [11] a RAIM algorithm is studied for detecting a fixed number of satellite failures. In [12] a backward search method is implemented. Another backward search is implemented in [13] for sensor networks. However, the standard literature on outlier detection usually considers the backward search as non robust for multiple outlier detection, since it is affected by masking (misidentification of outliers) and swamping (identification of good measurements as outliers) [14]. Some examples of robust algorithms can be found in GPS bibliography, as Huber M-estimation [15], and for LPS systems [16, 17]. Huber M-estimation has convergence problems when a good initial estimation is not available, whereas the robust methods presented in [16] and [17] do not take into account the geometry, with decreased robustness when a temperature measurement is not available.

In this paper a new robust method called RoPEUS (Robust Position Estimation in Ultrasound Systems) based on the MM estimator [18] is presented. The MM estimator is computed in three stages: an initial regression estimate with a high breakdown point, an M-estimate of the error scale, and a refining M-estimate using a redescending function. The use of two M-estimates inside the algorithm is the reason for calling them MM estimators. The low efficiency of high breakdown point robust algorithms is complemented in MM estimation with a robust final estimation step with high efficiency, but maintaining the high breakdown point of the initial estimation. The MM estimator has become very popular for this reason [19]. Three main modifications are added to the standard MM algorithm: 1) an intermediate checking of the high breakdown point estimator solutions is built into the algorithm, to make it robust to bad geometries; 2) the scale for the high efficiency algorithm is obtained from the off-line calculation of the standard deviation of the measurements using real data, and a geometry parameter of the initial estimation; and 3) a final check of the solution is added for taking into account the convergence of the algorithm to local minima. This algorithm is tested for static positioning in the 3D-LOCUS ultrasound localization system [20] in an ideal environment without obstacles. These measurements are corrupted with typical outlying patterns to numerically evaluate the algorithm performance with respect to the standard Parity Space (PS) algorithm.

The next section will present the background mathematics needed to introduce the algorithms employed, including basic regression concepts, the least squares regression algorithm and the corresponding equations in the ultrasonic system. The following section introduces the PS algorithm. Section 4. presents the RoPEUS algorithm. The evaluation of both algorithms is designed in Section 5. and the results presented in Section 6. Finally, Section 7. contains discussion and conclusions.

## 2. Mathematical Background

Although the equations defining the position estimation problem from measured ranges are nonlinear, most algorithms rely on the linearized equations to find the solution. The nomenclature employed in regression analysis and the basic regression algorithm employed to solve the linearized equations is introduced in this section. Its relation to the position estimation equations is presented next.

### 2.1. Regression Basics

The purpose of regression analysis is to fit equations to observed variables [21]. The basic equations that define the univariate regression problem are:

(1) $$y_{i}=g({\text{x}}_{i},\beta )+e_{i}\;\text{for}\;i=1,\ldots ,n,$$

where *n* is the sample size. The variables x*_i_* are the *independent variables* or *carriers*, whereas the variable *y_i_* is the *dependent variable* also referred as to *observations*. The vector *β*, with *p* unknown parameters,

(2) $$\beta =\left[\begin{array}{c} \beta_{1}\\ \beta_{2}\\ \vdots \\ \beta_{p} \end{array}\right]$$

(vectors and matrices will be denoted by boldface), has to be estimated from the data X and y, with:

(3) $$\text{X}=\left[\begin{array}{c} {\text{x}}_{1}\\ {\text{x}}_{2}\\ \vdots \\ {\text{x}}_{n} \end{array}\right]=\left[\begin{array}{cccc} x_{11} & x_{12} & \cdots & x_{1m}\\ x_{21} & x_{22} & \cdots & x_{2m}\\ \vdots & \vdots & \ddots & \vdots \\ x_{n1} & x_{n2} & \cdots & x_{nm} \end{array}\right]$$

where *m* is the dimension of the carriers. Applying a regression estimator to such a data set yields:

(4) $$\hat{\beta }=\left[\begin{array}{c} {\hat{\beta }}_{1}\\ {\hat{\beta }}_{2}\\ \vdots \\ {\hat{\beta }}_{p} \end{array}\right]$$

where the estimates (*β̂*_1_,*β̂*_2_,…,*β̂_p_*) are called the *estimated regression coefficients*. Applying Equation 1 to these coefficients yields:

(5) $${\hat{y}}_{i}=g\left({\text{x}}_{i},\hat{\beta }\right)\;\text{for}\;i=1,\ldots ,n$$

with *ŷ_i_* the *predicted* or *fitted* value of *y_i_*. The *residual* or *measurement residual r_i_* of the *i*th equation is the difference between what is actually observed and what is fitted:

(6) $$r_{i}\left(\hat{\beta }\right)=y_{i}-{\hat{y}}_{i}.$$

### 2.2. LS Regression Algorithms

It is usually considered that the error term *e_i_* of Equation 1 is normally distributed with equal variance *σ*^2^ (*e_i_* ∼ *N* (0, *σ*^2^)). In this case, the maximum likelihood estimator of the regression coefficients is the *least squares* (LS) estimator, defined as:

(7) $$\text{arg}\underset{\hat{\beta }}{\min}\left\{L\left(\hat{\beta }\right)\right\}$$

with:

(8) $$L\left(\hat{\beta }\right)=\frac{1}{2}\sum_{i=1}^{n}{\left(r_{i}\left(\hat{\beta }\right)\right)}^{2}=\frac{1}{2}{\left\|r\left(\hat{\beta }\right)\right\|}^{2}=\frac{1}{2}\text{r}{\left(\hat{\beta }\right)}^{T}\text{r}\left(\hat{\beta }\right).$$

One option for obtaining the regression coefficients satisfying Equation 7 is through linearization and iterative estimation of *β̂*. Linearizing Equation 1 using a Taylor series around the approximate regression coefficients (*β̂*_1_,*β̂*_2_,…,*β̂*_p_), we obtain:

(9) $$\begin{array}{r} \text{g}(\hat{\beta }+\Delta \hat{\beta })=y≃\text{g}\left(\hat{\beta }\right)+J\left(\hat{\beta }\right)\Delta \hat{\beta }\Rightarrow \text{r}\left(\hat{\beta }\right)=J\left(\hat{\beta }\right)\Delta \hat{\beta }\Rightarrow \\ \Rightarrow \Delta \hat{\beta }={\left(J{\left(\hat{\beta }\right)}^{T}J\left(\hat{\beta }\right)\right)}^{-1}J{\left(\hat{\beta }\right)}^{T}\text{r}\left(\hat{\beta }\right) \end{array}$$

where *J* is the Jacobian matrix and Δ*β̂* is the actualization step. The Gauss Newton method provides the same step, having the sign of the Jacobian matrix inverted.

The Gauss-Newton method has several drawbacks, the unguaranteed convergence being the most important. However, the Levenberg-Marquardt method guarantees convergence. Its step is defined by the following equation:

(10) $$\Delta \hat{\beta }={\left(J{\left(\hat{\beta }\right)}^{T}J\left(\hat{\beta }\right)+\mu I_{p}\right)}^{-1}J{\left(\hat{\beta }\right)}^{T}\text{r}\left(\hat{\beta }\right)$$

with *μ* ≥ 0. The parameter *μ* ensures the algorithm's convergence, since for all *μ* > 0 the coefficient matrix of Equation 10 is positive definite. This parameter starts with a predefined value *μ_init_* and changes in every iteration of the algorithm. When it is large, the step is close to the gradient direction, which is good if the current estimation is far from the solution. If it is small, the step will be close to the Gauss-Newton step, which is good for the final steps of the iteration process when the estimation is close to the solution. Consequently, we used this in this paper. A detailed implementation of the algorithm is presented in [22].

### 2.3. Trilateration equations in ultrasound location systems

The usual formulation for ultrasound LPS trilateration considers a known velocity of sound that is obtained through the measurement of the ambient temperature. This approach induces errors since the velocity of sound depends on more parameters (such as humidity), and the temperature is not uniform in indoor environments.

The equations that define the trilateration problem, when the velocity of sound is considered unknown, are:

(11) $$ToF_{i}=\frac{\sqrt{{\left(x_{b_{i}}-x_{u}\right)}^{2}+{\left(y_{b_{i}}-y_{u}\right)}^{2}+{\left(z_{b_{i}-z_{u}}\right)}^{2}}}{V_{s}}+e_{i}\;\text{for}\;i=1,\ldots ,n,$$

where *ToF_i_* is the measured time of flight between the user and the beacon “*i*”, (*x_b_i__, y_b_i__, z_b_i__*) is the known position of the beacon “*i*”, (*x_u_, y_u_, z_u_*) is the unknown user position, *V_s_* is the unknown sound velocity, *e_i_* is the error term, and *n* is the sample size.

The variables (*x_b_i__, y_b_i__, z_b_i__*) are the *carriers* (x*_i_*), *ToF_i_* are the *observations* for the dependent variable (*y_i_*), and the unknown parameters are:

(12) $$\beta =\left[\begin{array}{c} \beta_{1}\\ \beta_{2}\\ \beta_{3}\\ \beta_{4} \end{array}\right]=\left[\begin{array}{c} x_{u}\\ y_{u}\\ z_{u}\\ V_{s} \end{array}\right]$$

Consequently, in this problem, the number of unknowns (*p*) is 4, and the dimension of the carriers (*m*) is 3.

The literature considering an unknown sound velocity is very scarce. Figueroa proposes the use of a specific linearized system in [23], but this approach is not appropriate when the beacons are arranged in a plane since the resulting matrices are singular. That is the reason for using the standard approach for nonlinear equations based on the Jacobian of these equations presented in the previous section. The resulting matrices are non singular with the beacons located in the same plane, and the Jacobian matrix is:

(13) $$J=\left[\begin{array}{cccc} \frac{x_{11}-{\hat{\beta }}_{1}}{{\hat{\beta }}_{4}^{2}{\hat{y}}_{1}} & \frac{x_{12}-{\hat{\beta }}_{2}}{{\hat{\beta }}_{4}^{2}{\hat{y}}_{1}} & \frac{x_{13}-{\hat{\beta }}_{3}}{{\hat{\beta }}_{4}^{2}{\hat{y}}_{1}} & \frac{{\hat{y}}_{1}}{{\hat{\beta }}_{4}}\\ \frac{x_{21}-{\hat{\beta }}_{1}}{{\hat{\beta }}_{4}^{2}{\hat{y}}_{2}} & \frac{x_{22}-{\hat{\beta }}_{2}}{{\hat{\beta }}_{4}^{2}{\hat{y}}_{2}} & \frac{x_{23}-{\hat{\beta }}_{3}}{{\hat{\beta }}_{4}^{2}{\hat{y}}_{2}} & \frac{{\hat{y}}_{2}}{{\hat{\beta }}_{4}}\\ \vdots & \vdots & \vdots & \vdots \\ \frac{x_{n1}-{\hat{\beta }}_{1}}{{\hat{\beta }}_{4}^{2}{\hat{y}}_{n}} & \frac{x_{n2}-{\hat{\beta }}_{2}}{{\hat{\beta }}_{4}^{2}{\hat{y}}_{n}} & \frac{x_{n3}-{\hat{\beta }}_{3}}{{\hat{\beta }}_{4}^{2}{\hat{y}}_{n}} & \frac{{\hat{y}}_{n}}{{\hat{\beta }}_{4}} \end{array}\right].$$

## 3. Parity Space Method for Outlier Detection

The most widespread method for detecting outlying measurements in GPS is the use of the parity vector [10, 24]. Using the matrix *J* obtained after the convergence of the Gauss-Newton or the Levenberg-Marquardt methods, we can obtain a matrix called P of dimensions (*n* − *p*) × *n* with rank *n* − *p*, such that P P*^T^* = I*_n_*_−_*_p_* and P *J* = 0. This matrix can be obtained through singular value decomposition [25] or QR factorization [26] of the matrix *J*. Using the QR factorization (*J* = QR) and dividing the matrix Q in two submatrices Q = [Q_1_, Q_2_], where Q_2_ has dimensions *n* × (*n* − *p*), the matrix P is obtained as $\text{P}={\text{Q}}_{2}^{T}$. The parity vector is then obtained from this matrix and the residual vector as:

(14) p=P r

To detect a faulty measurement, the vector p can be employed, and also its transformation from the parity space to the measurement space, that employs the matrix S = P*^T^*P = I − *J* (*J^T^ J*)^−1^ *J^T^* (with rank *n* − *p* and having the property of being idempotent: S^2^ = S), obtaining the faulty vector:

(15) f=S r.

A decision variable for detecting a faulty measurement can be calculated from these vectors; we will employ *D* = p*^T^*p = f*^T^*f. When it reaches a threshold (*T*), we will deem that there is an erroneous measurement. The detection threshold (T) is a function of the false alarm probability (*P_FA_*) defined as the probability of the decision variable being above the threshold without any measurement error. From this value, that is supplied to the algorithm, we can calculate the value of *T* as [10]:

(16) $$P_{FA}=1-\sigma^{2}\chi_{n-p}^{2}(T)\Rightarrow T=\chi_{n-p,(1-P_{FA})/\sigma^{2}}^{2}$$

If an error is detected (*D* > *T*), the maximum value of $f_{i}^{2}/S_{ii}$ is calculated. The index *i* of this maximum identify the faulty measurement, which is deleted.

## 4. RoPEUS Algorithm

**Figure 1.**
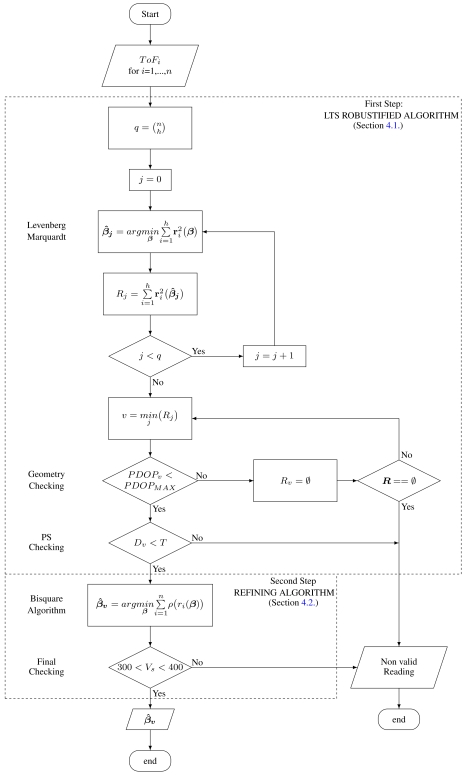
Flowchart of the RoPEUS algorithm.

The RoPEUS algorithm, proposed by the authors of this paper, is inspired in the MM estimator of Yohai [18]. The MM estimator is divided into three stages: an initial regression estimate with a high breakdown point algorithm, an M-estimate of the error scale, and a refining M-estimate with a redescending function. The RoPEUS algorithm follows the same strategy with the exception that the error scale (estimated standard deviation of the measurement error) is performed off-line, due to the small number of measurement employed in a single estimation. With this modification, the RoPEUS algorithm consist of two steps: a high breakdown point estimation step and a refining step. The high breakdown algorithm employed is based on the Least Trimmed Squares (LTS) estimator, which is modified to make it robust to bad geometries and to detect erroneous results. The refining M-estimate considered is the bisquare estimator, that employs the error scale calculated off-line. Figure 1 shows a flow chart of the proposed algorithm.

### 4.1. First step: LTS Robustified Method

Standard least-squares regression diagnostic methods, such as Parity Space, are affected by two related phenomenons: masking (misidentification of outliers) and swamping (identification of good measurements as outliers) [14]. These effects make the use of robust estimators for position calculation more appropriate.

The LTS estimator is the value that minimizes the expression:

(17) $$\text{arg}\underset{\hat{\beta }}{\min}\left\{\sum_{i=1}^{h}{\text{r}}_{\left(i\right)}^{2}\left(\hat{\beta }\right)\right\}$$

where ${\text{r}}_{\left(i\right)}^{2}(\hat{\beta })$ are the squared residuals (r (*β̂*)) written in ascending order (brackets in the subindex denote ordered increasing values). Since the minimization considers the *h* (< *n*) smallest squared residuals, it will be inmune to *n* − *h* failing measurements.

For its calculation, we compute the estimated position using the Levenberg-Marquardt algorithm for all combinations of the *n* measurements taken *h* at a time; as such we get $q=\left(\begin{array}{c} n\\ h \end{array}\right)=\frac{n!}{h!(n-h)!}$ possible solutions. Then, for each solution, we compute the corresponding *n* residuals, arrange them in ascending order, and sum the *h* smallest squared residuals. We can store these results in a vector *R* with *q* elements. The regression coefficients associated with the index *v* of the minimum of this vector (*β̂_V_*) are the solutions of the algorithm [21].

The LTS algorithm in its basic form does not take into account the estimated precision of the result. This makes the system prone to reduce the accuracy of the algorithm, mainly when any or several subgroups of satellites form a bad geometry. The algorithm proposed in this paper takes into account the geometry using an estimation of its goodness to reject those subgroups having a bad geometry.

The standard deviation of the estimation error is related to the standard deviation of the measurements by the square root of the diagonal elements of the matrix *DOP* = (*J^T^ J*)^−1^ [27]. These values and their combination are usually referred in GPS bibliography as Geometric Dilution of Precision parameters (GDOP [28]). This name is based on the fact that the Jacobian matrix depends on the existing geometry of the beacons with regard to the user position.

The parameter selected for rejecting bad geometries is the Position Dilution of Precision (PDOP) that relates the positioning Root Mean Square (RMS) error with the standard deviation of ToF measurements. Although this parameter is dimensionless in the GPS system, it will have units of [m/s] in our algorithm since the positioning error is measured in meters and the time of flight is measured in seconds. The PDOP parameter is calculated from the *DOP* matrix above, being:

(18) $$\text{PDOP}=\sqrt{\sum_{i=1}^{3}{\text{DOP}}_{ii}}\;[m/s]$$

This parameter can be computed for the *q* possible solutions of the LTS algorithm calculated above. Those surpassing a maximum predefined PDOP limit (*PDOP_MAX_*) are rejected and not taken into account for the final LTS solution. Notice that it is not necessary to calculate this parameter for all the possible solutions (which increase the calculations) but only for those candidate solutions (*β̂_v_*) with smaller values in the vector *R*.

Since this algorithm does not take into account the existence of too many erroneous measurements (more than *n* − *h*), a parity space checking of the final solution is made. If an outlier is detected, the solution is flagged as “non valid reading”.

### 4.2. Second Step: Refining Step

The second step needs the off-line calculation of the ToF measurement error variance, which is performed using a large number of position estimations previous to the algorithm application. Using the RMS error of these position estimations and its averaged PDOP, the estimated standard deviation of the measurements will be:

(19) $$\sigma =\frac{\text{RMS}}{{\text{PDOP}}_{\text{MEAN}}}\;[s]$$

This value will take into account both the standard deviation and bias of the ToF measurements. The scale of the residuals will be estimated from *σ* and the PDOP parameter on every position determination using the following formula:

(20) $$\sigma_{r}=\frac{\sigma \text{PDOP}}{{\hat{\beta }}_{4}}\;[s]$$

This will be the value employed in this refining step as the scale.

M-estimation is based on the minimization of a function of the residuals, aiming at a result not influenced by outliers. The M-estimation function considered for attaining higher efficiency is the bisquare (also called biweight) function, which is a redescending estimate. This function is defined as:

(21) $$\rho (r_{i})=\{\begin{array}{ccc} 1-{\left[1-{\left(\frac{r_{i}}{\sigma_{r}k}\right)}^{2}\right]}^{3} & \text{if} & \left|r_{i}\right|\leq \sigma_{r}k\\ 1 & \text{if} & \left|r_{i}\right|>\sigma_{r}k \end{array}$$

The value of *k* will determine the efficiency of the algorithm. Table 1 shows the values of the parameter *k* for prescribed efficiencies of the algorithm [19].

**Figure 2.**
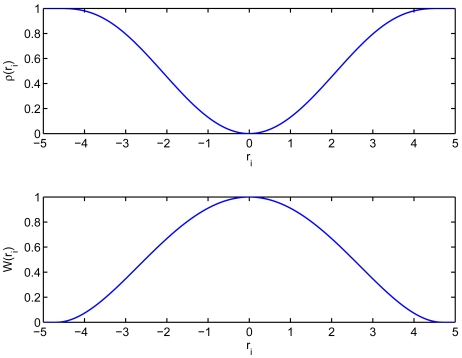
Biweight function and its corresponding weighting function.

The algorithm implemented for minimizing this function of the residuals is a weighted least squares. The weighting function corresponding to the biweight is:

(22) $$W(r_{i})={\left[1-{\left(\frac{r_{i}}{\sigma_{r}k}\right)}^{2}\right]}^{2}\text{I}\left(\left|r_{i}\right|\leq \sigma_{r}k\right)$$

where I(.) is the indicator function, whose value is 1 when the argument is fulfilled and 0 otherwise. Figure 2 shows both the *biweight* function (Equation 21) and its corresponding weighting function (Equation 22) with *σ_r_* = 1 and *k* = 4.68.

A final check of the result is added at the end of the algorithm, to discard those estimations that has converge to *impossible* values. It is checked whether the estimated velocity of sound is within a reasonable value. Notice that this range do not depend on any temperature measurement.

## 5. Outlier Characterization and Test Design

A numerical evaluation of the algorithm has been made by controlling the outliers pattern present in the measurements. Consequently, the data considered in the evaluation is composed of real measurements and typical outlying patterns. This approach also checks whether the estimated value of sigma is correct under real measurements.

### 5.1. Typical outliers in ultrasound LPS

**Figure 3.**
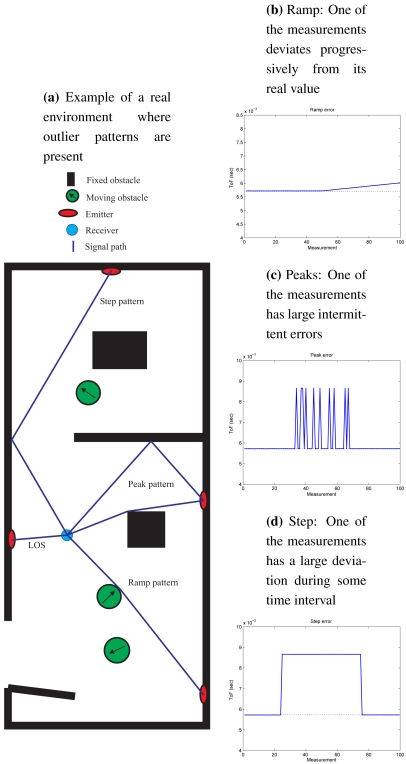
Outlying patterns considered in the evaluation of the algorithm.

The outlying patterns in ultrasound range measurements are basically of three types: ramp, peak and step. These patterns are presented in Figure 3, applied to a real ToF measurement, to show the importance of these errors with respect to the actual value. Figure 3a shows an example of a real environment where these outlier patterns would be present.

Figure 3b shows a typical outlying ramp pattern: one of the measurements deviates progressively from its true value. This pattern is produced when a moving obstacle partially occludes the direct signal path. The signal is propagated by refraction on the obstacle surface. The maximum deviation is not large, making difficult its detection.

The peaks pattern showed in Figure 3c is characterized by large intermittent outliers. These errors are usually produced when the power of the signal refracted in an obstacle is very low and there is multipath present. The system is not able to distinguish which signal is the correct one. An intermittent change between two different times of flight is produced.

When the direct signal path is completely occluded an outlying step occurs (see Figure 3d). It is characterized by a continuous large error since the only detected signal is coming from an indirect path.

### 5.2. Test Design

The RoPEUS and PS algorithms performance will be evaluated using real measurements obtained in an ideal environment (without obstacles) with the 3D-LOCUS ultrasound local positioning system [20]. This system consist of a network of seven fixed nodes located at known positions and a mobile node whose position is calculated. Every node has one emitter and one receiver of ultrasonic signals, being able to work as a centralized system (the mobile node emits and the fixed nodes receive the ultrasonic signal), private (the mobile node receives the ultrasonic signals emitted from the fixed nodes), or bidirectional (signals are sent to and from the mobile node). Original measurements are borrowed from [9] where the system performance was evaluated, taking those belonging to the bidirectional mode in a windy environment and using Time Division Multiple Access (TDMA). These measurements are taken with seven fixed nodes located in the ceiling of a fixed structure forming and hexagon with one of them in its center. The mobile node is located in 22 fixed positions taking 100 individual measurements in each of them.

The original measurements from [9] will be corrupted in this paper with the outlier patterns presented in the previous section following Table 2. Four different cases are considered: 1) Original measurements; 2) Step pattern; 3) Ramp pattern; 4) Step and peak patterns simultaneously in two different beacons. The step outlier is applied to every measurement of one of the beacons with a value of 2,941 *μ*s (1,000 mm at 340 m/s). The outlying ramp is also applied to every measurement of one of the beacons and is repeated on every single test point with a maximum value of 294 *μ*s (100 mm at 340 m/s). The peaks pattern is applied randomly to a 25% of the measurements (550) in a different beacon with a value of 2,941 *μ*s. Notice that the peaks pattern applied to a 100% of the measurements would be the same as the step pattern.

### 5.3. Design of the algorithms

The main configuration parameters employed in the evaluation of the PS and RoPEUS algorithms are presented in Table 3.

The standard deviation of ToF measurements is calculated off-line using Equation 19. Using the RMS error and the mean PDOP of the original 2,200 position determination presented in [9], results in *σ* = 3.444 *μ*s for bidirectional TDMA with wind. This value will be used in both algorithms the parity space and the refining step of RoPEUS (in the bisquare estimator).

The false alarm value in the parity space algorithm is fixed to 1%. The same algorithm is implemented at the end of the LTS robustified algorithm for rejecting erroneous measurements; the value employed is 0.1% in this case. We take a smaller value in the latter case since it must only detect big errors in the estimation.

Since there are seven measurements available in every position estimation (*n* = 7), a maximum of two erroneous measurements can be detected (having in this case five correct measurements which implies one redundant measurement since we estimate four parameters). Therefore, if the parity space algorithm detects three erroneous measurements the estimation will be flagged as non valid. The implication in the LTS algorithm is that *h* equals 5 in the Equation 17.

The PDOP parameter employed in the LTS algorithm for rejecting bad geometries will be fixed to 2,000 m/s. An intuitive approach to the significance of this value can be obtained dividing it by a standard velocity of sound which approximately results in the amplification rate of the distance measurement errors due to the geometry (with the real velocity of sound it would result in the exact amplification of the standard deviation of the distance measurements). For instance, with a velocity of sound of 340 m/s the amplification ratio would be 5.88.

A final check of the results is added for deleting anomalous values, that occur when the algorithm converges to local minima. This check consists in flagging as non valid measurements those with an estimated velocity of sound outside the 300-400 m/s range.

Finally, the starting point of the algorithms is located 0.5 meters below the centroid of the beacons (approximately the center of the hexagon) and a velocity of sound of 320 m/s is considered as an initial seed.

## 6. Results

**Figure 4.**
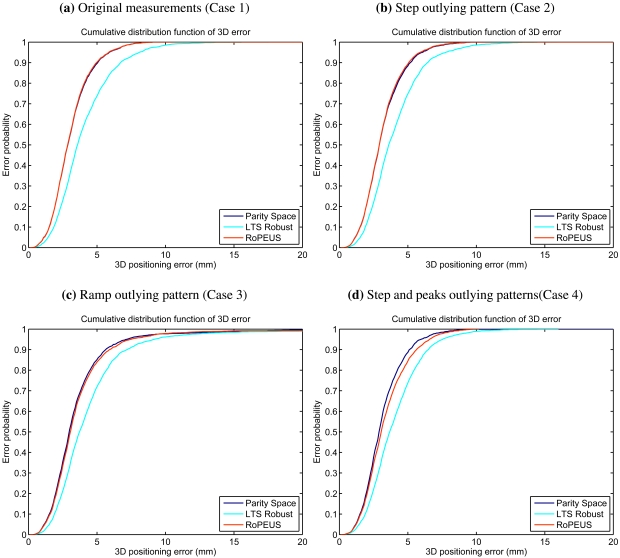
Cumulative distribution function obtained when applying the PS and RoPEUS algorithms to the four cases studied (see Table 2).

The results obtained are presented in Figure 4 and Table 4, offering a qualitative and quantitative comparison of the algorithms performance. Figure 4 includes the results obtained after the first step of the RoPEUS algorithm (LTS Robust). This step give robustness under multiple outliers to the global algorithm, while the second step, whose results correspond to the RoPEUS algorithm, improves the accuracy (as can be seen in Figures 4a–4d). The numerical results extracted from the algorithms (Table 4) are the RMS of the positioning error, maximum and 95% confidence level errors, the simulation time, and the number of non valid measurements.

The evaluation of the accuracy can be extracted from the numerical errors and the cumulative distribution functions. They show that both algorithms get very similar results, being slightly better for the PS algorithm in the last case (see Figure 4d). The worst case outlying pattern is the ramp error, where both algorithms get a RMS error below 4.8 mm and a 95% level below 7.4 mm.

The robustness of the algorithms is evaluated in terms of non valid measurements, which is clearly represented in the numerical results. The lack of robustness of the parity space algorithm is clearly shown when two outliers are present (step and peak errors, whose results are presented in Table 4), where the number of non valid estimations is approximately equal to the number of evaluations with two simultaneous erroneous measurements. It is observed in Table 4 that the original measurements sample without contamination contains 1 erroneous measurement that is detected by both algorithms.

The simulations have been performed in a Pentium 4 processor at 3.20 GHz with the algorithms implemented in Matlab. The computational load of the algorithms is reflected in the total time employed for the 2,200 estimations, shown in Table 4. The RoPEUS algorithm takes about five times the time employed by the PS algorithm. The maximum computation time correspond to the case with two simultaneous outliers, where the RoPEUS algorithm lasts for about 115 seconds that correspond to a maximum actualization rate of the position of 19 Hz.

## 7. Discussion and Conclusions

A new algorithm called RoPEUS, based on the MM estimator, has been proposed and tested in this article. Its robustness, accuracy and computational load makes it suitable for being implemented in any ultrasound positioning system.

The general structure of the algorithm based on two steps (robust and refining steps) is borrowed from the MM algorithm. The scale estimation step considered in the MM algorithm is omitted and calculated off-line due to the reduced number of measurements available, which is usually the case in global and local positioning systems.

If this algorithm would be applied to a system with more measurements available (more fixed beacons in our example), the on-line estimation of the scale could improve the solution. However, the improvement is not secure and the number of measurements necessary for a reliable result remains to be studied; whence, we recommend to make the scale calculation in the way it has been presented. However, an increment in the number of beacons could result in an infeasible computation time in the LTS robust step. This could be overcome with the consideration of only a random selection of all the possible combinations of the *n* measurements [19]. Anyway, we propose to select the seven smaller ToF measurements, when we scale the 3D-LOCUS system with more fixed beacons, to avoid the increased computation time.

The LTS robustified algorithm discards those solutions where the numerical stability of the equations is compromised, and allows for the existence of a large error. This approach, compared to the one presented in [17], has the advantage of avoiding the necessity of determining an approximate range for the velocity of sound (although the range employed in this article was coarse, the result depended on the width of this range). The PS checking is included for detecting those estimations where more than two erroneous measurements are present. The result then obtained is just an intermediate estimation, that is refined with the bisquare estimator requiring a “good” starting point.

The implementation of the refining biweight estimator enables the LTS algorithm to have a bigger error, consequently the DOP limit does not need to be very tight. This algorithm achieves a robust final improvement of the solution with a slight increase of the computation time. It automatically rejects or takes into account measurements discarded by the LTS algorithm, without having a deterministic number of influential data.

Although the velocity of sound estimation presents better accuracy than the traditional calculation from the temperature data, it presents the problem of local minimums in a few evaluations with outliers. It has been observed that these minimums usually fall far from the real solution, presenting anomalous velocity estimations. This is the reason for adding a final check that detects whether the value of the estimated velocity of sound is reasonable or not.

The robustness of the RoPEUS algorithm carries an important increment in the computational load. However, it is not excessive for 3D-LOCUS since the update rate of 19 Hz enabled by the algorithm is larger than the actual update rate of the 3D-LOCUS system, with a maximum of 10 Hz in CDMA mode (tested with four beacons) and around 2 Hz in TDMA bidirectional mode [9]. Consequently, this algorithm can be implemented in the central computer that is connected to the 3D-LOCUS system for real time operation. We also consider that an optimized implementation could be made to run this algorithm in the central node, or alternatively, distributing the needed computation among the sensor nodes of the 3D-LOCUS system. However, RoPEUS cannot be implemented in other nodes with severe battery restrictions due to its computational demands. In such processing and battery-limited sensor networks a centralized implementation must be made.

The result of the RoPEUS algorithm is an accurate position estimation that does not rely on any temperature measurement or approximate range, is robust under two simultaneous outliers (using seven beacons), and has an appropriate position update rate that does not slow down the system. This results in an increase of the system availability, improved reliability and improved general performance.

## Figures and Tables

**Table 1.** Efficiency of the bisquare algorithm for several values of the parameter *k* [19].

eff.	0.80	0.85	0.90	0.95
*k*	3.14	3.44	3.88	4.68

**Table 2.** Outlying patterns added to the original measurements to evaluate the RoPEUS algorithm performance.

Cases under consideration	Type of errors added to original measurements		

	Ramp	Peak	Step

Case 1			
Case 2			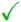
Case 3	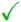		
Case 4		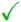	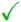

**Table 3.** Main configuration parameters employed in the simulations.

Algorithm		Parameter	Value

PS		Base algorithm	Levenberg-Marquardt
		Max iterations	25
		*μ_init_*	10^−6^
		*σ*	3.444 *μ*s
		*P_FA_*	1%
		Max errors	2

RoPEUS	LTS robust	Base algorithm	Levenberg-Marquardt
		Max iterations	25
		*μ_init_*	10^−6^
		*σ*	3.444 *μ*s
		*P_FA_*	0.1%
		h	5
		*PDOP_MAX_*	2,000 m/s

	MM (Bisquare)	Base algorithm	Weighted Least Squares
		Max iterations	25
		*σ*	3.444 *μ*s
		Efficiency	0.95%
		k	4.68

**Table 4.** Numerical results obtained when applying the PS and RoPEUS algorithm to the four cases studied (see Table 2).

**Outlying pattern**	**Algorithm**	**RMS(mm)**	**95%(mm)**	**Max(mm)**	**Time(sec)**	**Non valid(#)**
Original measurements	Parity Space	**3.4132**	5.7	9.0866	12.3071	**1**
	RoPEUS	**3.3916**	5.7	9.0889	86.2593	**1**
Step	Parity Space	**3.5039**	5.9	11.7279	19.8974	**1**
	RoPEUS	**3.4532**	5.7	9.5744	112.6032	**1**
Ramp	Parity Space	**4.4343**	7.0	34.7512	19.1777	**3**
	RoPEUS	**4.7735**	7.3	46.628	84.8579	**1**
Step and peaks	Parity Space	**3.4912**	5.8	11.7279	22.2398	**551**
	RoPEUS	**3.7456**	6.4	10.1281	115.4485	**15**
